# A prototype-based resonance model of rhythm categorization

**DOI:** 10.1068/i0665

**Published:** 2014-11-04

**Authors:** Rasmus Bååth, Erik Lagerstedt, Peter Gärdenfors

**Affiliations:** Lund University Cognitive Science, Lund University, LUX, Lund, Sweden; e-mail: rasmus.baath@lucs.lu.se; Lund University Cognitive Science, Lund University, LUX, Lund, Sweden; e-mail: lagerstedt.erik@gmail.com; Lund University Cognitive Science, Lund University, LUX, Lund, Sweden; e-mail: peter.gardenfors@lucs.lu.se

**Keywords:** categorical perception, rhythm perception, computational modeling, music perception, dynamical systems, resonance theory

## Abstract

Categorization of rhythmic patterns is prevalent in musical practice, an example of this being the transcription of (possibly not strictly metrical) music into musical notation. In this article we implement a dynamical systems' model of rhythm categorization based on the resonance theory of rhythm perception developed by Large (2010). This model is used to simulate the categorical choices of participants in two experiments of Desain and Honing (2003). The model accurately replicates the experimental data. Our results support resonance theory as a viable model of rhythm perception and show that by viewing rhythm perception as a dynamical system it is possible to model central properties of rhythm categorization.

## 1 Introduction

Music is a nondiscrete art form since it exists in the auditory domain where differences in rhythm, pitch, and timbre are continuous. However, when this continuous domain is described, discrete categories are often used, e.g., pitch is categorized as scale notes. Rhythm is also the subject of categorization, and an example of this is the transcription of the rhythm of a piece of music into musical notation. This process constitutes a categorization, as the number of ways to notate a rhythmic sequence is finite and different rhythmic sequences can be notated in the same way. Desain and Honing (2003) showed in two experiments that listeners reliably experienced rhythms as belonging to rhythmic categories and that categorizations were strongly influenced when the listeners were primed with a metric beat before hearing a rhythm. Furthermore, participants agreed to a large degree on which rhythms belonged to what category. Just as with categorization of other kinds of stimuli (c.f. Jäger, 2010, on color categories), the categories were roughly convex with respect to a temporal performance space as discussed in Section 3 (Gärdenfors, 2000). Honing (2013, p. 379) concludes that: “It is puzzling, however, that although meter was shown to exert a strong influence on the recognition of rhythm […] existing computational models of meter can explain this phenomenon only to a small extent.”

In this article we show that an oscillation-based, *resonance theory* model of rhythm perception (Large, 1996, 2010) can replicate many of the findings of Desain and Honing (2003). Although oscillator models have previously been applied to many different aspects of music perception (e.g., Angelis, Holland, Upton, & Clayton, 2013; Large, 1996, 2010), such models have not previously, to our knowledge, been applied to categorical perception. Our results support the notion that resonance theory is a viable model of rhythm perception and show that by viewing rhythm perception as a dynamical system it is indeed possible to model the properties of categorical rhythm perception. Furthermore, these results suggest that oscillator models can be applied to other types of categorical perception, for example, pitch perception and vowel perception.

### Resonance Theory and Rhythm Perception

In the field of music perception, *rhythm* refers to a temporal pattern of sound onsets (McAuley, 2010). A rhythm in this sense does not have to be periodic or recurrent. This is in contrast with how that word is used in other fields (cf. circadian rhythm or delta rhythm). A related concept that does involve periodicity is *beat*. When listening to a piece of music, a common response is to move one's body with a perceived periodic pulse (Snyder & Krumhansl, 2001), that pulse being the beat of the corresponding piece of music. It is common that not all beats in a piece of music are perceived as being equally accented (Palmer & Krumhansl, 1987) and a periodically recurring pattern of strong and weak beats is called a *meter*. For example, a duple meter would imply that every second beat is perceived as having a stronger accent while every third beat is perceived as having a stronger accent in the case of a triple meter. Rhythm perception and the ability to entrain to a musical beat was long thought to be uniquely human and, while it has recently been shown that some vocal mimicking species are, to some degree, able to move in synchrony with a beat (Schachner, Brady, Pepperberg, & Hauser, 2009), humans are still unique in their aptitude for rhythmic processing. Already infants have been shown to have a sense of rhythm (Honing, Ladinig, Háden, & Winkler, 2009) and there exists only one documented case of “beat deafness” (Phillips-Silver et al., 2011), that is, the inability to reliably synchronize to a musical beat.

Modeling of human timing and rhythm perception has a long history, one influential model being that described by Wing and Kristofferson (1973), which is based on an information theoretic perspective. Like many such models (cf. Repp, 2005), it models a participant's behavior in situations where isochronous timing responses are being elicited. An alternative to the information theoretic perspective is to take a dynamical systems perspective and model time and rhythm perception as an emergent, dynamic phenomenon. A number of models of this kind have been proposed (e.g., Large, 1996; Todd, O'Boyle, & Lee, 1999; van Noorden & Moelants, 1999). Here, the term *resonance theory* (cf. Large, 2010) will be used to refer to such models. The general idea of resonance theory is that an external auditory rhythm can be represented by the amplitude of internal oscillatory units. These oscillatory units are coupled to the external rhythm and are by definition periodic while the external rhythm does not have to be periodic. Given a rhythm sequence as input, the basic output of a resonance theory model, or resonance model for short, is the amplitude response of the oscillators over time. Resonance theory does not dictate a specific model but rather incorporates a number of related models which all can be considered dynamical system models.

Resonance theory provides a compelling framework since it is biologically plausible, has a solid base in dynamical systems theory and is able to model many aspects of meter and rhythm perception (Angelis et al., 2013; Large, 2000). A number of neuroimaging studies have shown connections between neural resonance and rhythm perception (e.g., Brochard, Abecasis, Potter, Ragot, & Drake, 2003; Fujioka, Trainor, Large, & Ross, 2012; Schaefer, Vlek, & Desain, 2011). One persuasive study that clearly showed that rhythm perception involves neural oscillatory activity is that of Nozaradan, Peretz, Missal, and Mouraux (2011). They found that playing a rhythmic beat to a participant elicited a sustained periodic neural response, as measured by EEG, that matched the frequencies of the beat.

As already noted, to our knowledge, resonance theory models have not previously been used to model categorical rhythm perception. One reason for this might be that while the amplitude response of the oscillators to a rhythm in the resonance model reflects, perhaps even represents, the rhythm sequence given as input to the system, it does not give rise to a categorization per se. That is, the state of the resonance model depends on the given rhythm sequence, but there is no single well defined set of states that can be said to constitute categories. Still, the state of the resonance model can be used as the basis of a categorical decision based on learned prototype states or a discrete partitioning of the system state space.

If the state of the resonance model is viewed as the basis for a categorical decision then two predictions regarding categorical rhythm perception can be made:

A More distinct states will facilitate categorization. Here we use *distinct state* to refer to a state of the resonance model where a small subset of oscillators has a strong amplitude response while most oscillators do not. This is in contrast to a nondistinct state where most oscillators have a similar amplitude response, that is, there are many competing signals and there is no clear single winning candidate among the categories. If some rhythmic sequence was known to result in a strongly distinctive state in a resonance model, then it could be predicted that a participant in an experimental categorization task would categorize that rhythm sequence consistently, and with more confidence than a rhythmic sequence known to result in a less distinctive state.
B Rhythm sequences resulting in similar states are categorized similarly. That is, different rhythm sequences resulting in similar states when used as the input to a resonance model should be categorized similarly by participants in an experimental task. Here similarity has to be defined using a similarity measure such as Euclidean distance or cosine similarity.

In order to test these predictions, data from the rhythm categorization task from the study by Desain and Honing (2003) were used.

### The Rhythm Categorization Study of Desain and Honing (2003)

**Figure 1.**
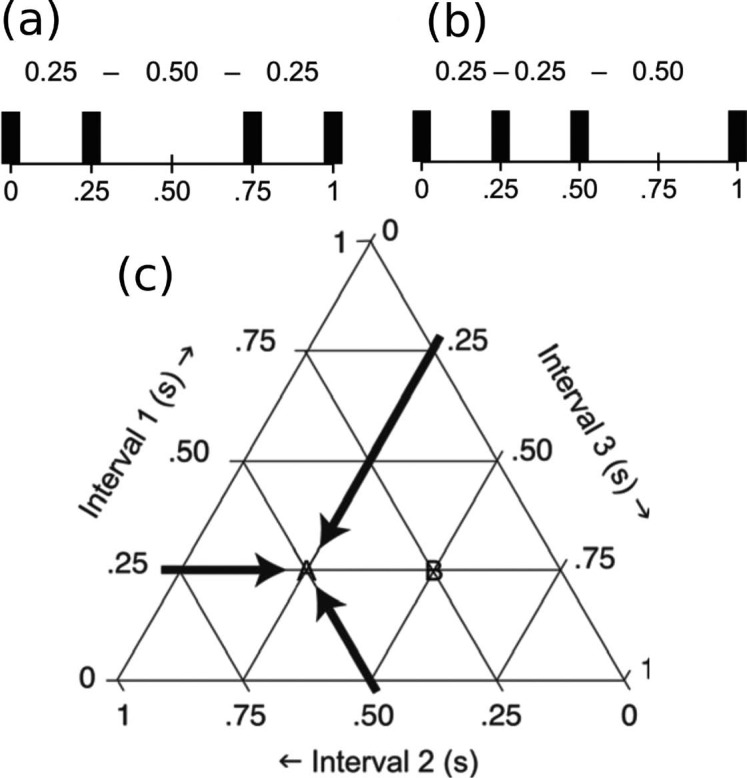
(a) and (b) show examples of two possible rhythms and their placement in the triangular performance space (c) defined by Desain and Honing (2003). All one second long, four sound rhythms can be represented as a point in this space. (From Honing, 2012 with permission).

**Figure 2.**
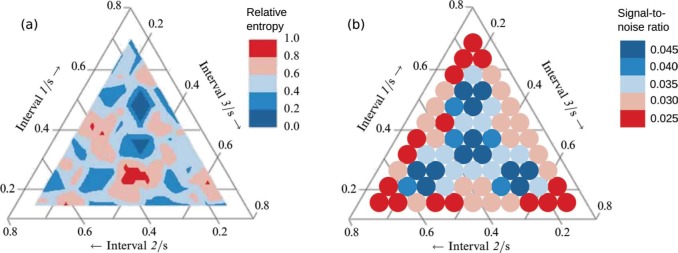
Maps over categorization consistency. (a) shows the relative entropy of the categorical choices for the single participant given the same rhythm sequences multiple times from Desain and Honing (2003, used with permission). The relative entropy is calculated as the Shannon entropy divided by the theoretical maximum attainable entropy. (b) shows the signal-to-noise measure calculated from the activation patterns generated by the resonance model.

Desain and Honing (2003) employed a novel paradigm where musically educated participants were asked to categorize 66 different rhythm sequences by transcribing them into common music notation. The sequences all lasted for one second and consisted of four tone onsets and were therefore uniquely determined by the three interonset intervals (IOI) between the tones. Two such possible sequences are shown in Figure 1a and 1b where a possible categorization of 1b could be ♪♪ ♩ (or 1-1-2 when written as an integer ratio). Any possible 1 s, four-tone rhythm sequence can be thought of as a point in a three dimensional triangular *performance space* that determines the lengths of the three IOIs as shown in Figure 1. The 66 rhythm sequences in Desain and Honing's experiment were constructed so that they evenly covered the area in the performance space with the constraint that no IOI would be shorter than 153 ms. The location of these sequences in the performance space can be seen in Figure 2b where each circle marks the position of one of the 66 sequences.

**Figure 3.**
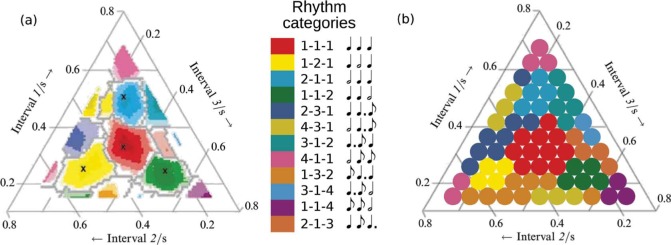
Categorization maps for (a) the experimental data from Desain and Honing (2003, used with permission) and (b) the resonance model. The transparent areas in (a) indicate areas where there was a large amount of disagreement between the participants.

In a first experiment, 29 highly trained musicians categorized the rhythm sequences and the result was that even though the rhythms occurred on a more or less continuous time scale, the participants tended to stick to a limited number of categories, with 1-1-1 being the most common. Twelve categories, all categories considered, stood out as being the most common and the location in performance space of these categories are shown in Figure 3a. One participant was presented with all 66 rhythm sequences at six different occasions and, as a measure of consistency, the entropy was calculated of her responses for each rhythm. These entropy values were mapped on to the performance space and the resulting entropy map is shown in Figure 2a.

In a second experiment, two metrical conditions were added. Duple meter versions of the rhythms were constructed by adding a repeated, 1-s long, two sound beats to the beginning of the rhythms, thus inducing a 2/4 meter feeling. Triple meter versions of the rhythms were similarly constructed by adding a three sound beat instead. This resulted in three different metrical conditions: The original no meter condition, a duple meter condition, and a triple meter condition. Maps over what categories the participants ascribed to the different rhythms, similar to the map shown in Figure 3a, were constructed (shown on p. 358 in Desain & Honing, 2003). A main finding was that the participants' categorization in the no meter condition was significantly more similar to the participants' categorization in the duple meter condition than in the triple meter condition.

For the purpose of the current study, data from Desain and Honing were downloaded from a web resource containing supplementary material (http://www.mcg.uva.nl/categorization). The data downloaded were the information regarding which of the 12 most common categories were most often ascribed to each of the 66 rhythm sequences for the no meter condition in experiment 1, and the duple and triple meter conditions in experiment 2. A rhythm sequence was excluded if none of the 12 most common categories was the most common for that specific rhythm. Information regarding the categorization entropy for the sole participant presented with the rhythms multiple times was calculated manually from Figure 2a.

### Resonance Theory and Rhythm Categorization

It is possible to test the two predictions from resonance theory concerning how rhythms are categorized by implementing a resonance model that consists of an array of oscillators (as in Large, 2000); see Section 2 for more details. We used the rhythm stimuli from Desain and Honing (2003) as input to such a model and compared the results with the experimental data using the methods outlined below. The output of a resonance model is a multidimensional time series with the same number of dimensions as the number of oscillators in the model. This high dimensional representation might be difficult to work with directly, however, and a more convenient representation is given by creating an *activation pattern*, *A*, by summing the amplitude responses of each oscillator over time, as in

(1) $$A_{i}=\sum_{t=t_{s}}^{t_{e}}a_{i,t},$$

where *ai*_*t*_ is the amplitude for oscillator *i* at time *t* while *t*_*s*_ and *t*_*e*_ are the start and end time steps for the summation. Before the resonance model is given any input it is in a resting state and it takes a number of time steps before the system is activated by the stimuli. Therefore, it is not necessarily desirable to sum over the whole extent of the duration of the rhythm sequence and an activation pattern created by summing over the later time steps may represent the rhythm sequence better than an activation pattern created by summing over all time steps. By considering the activation pattern of a resonance model as a point in an *n*-dimensional space, *n* being the number of oscillatory units, this space can be partitioned into regions corresponding to rhythm categories and used to produce categorical decisions (following the general model of concepts from Gärdenfors, 2000). Given that entire equivalence classes of different spectra can give rise to the same color perception, the potential relation between the activation pattern of a resonance model and such a rhythm categorization is analogous to the relation between the hue, saturation, and lightness of a color percept and a color categorization. That is, a color percept can similarly be viewed as a point in a three-dimensional space with dimensions hue, saturation, and lightness and this space can be partitioned into regions, each representing a color category, and used to produce categorical color decisions.

Prediction (A) implies that rhythm sequences resulting in distinctive states (in the sense discussed earlier) in a resonance model should be the sequences that are categorized more consistently. In Desain and Honing's data, a measure of consistency is the categorization entropy for the participant presented with the rhythm sequences multiple times. The prediction is that this measure of consistency is correlated with a measure of distinctness of the state of a resonance model. Signal-to-noise ratio is a common measure of distinctness of a signal and a modified version of this measure can be used to quantify the distinctness of the state of a resonance model. For a resonance model that has been given a rhythm sequence as input, the activation pattern is first calculated according to Equation (1). In this activation pattern, the signal *A*_*s*_ is defined as being the *A*_*i*_ with the highest amplitude. The signal-to-noise ratio is then defined as:

(2) $$\mathrm{SNR}=\frac{A_{s}}{\sum_{i=1}^{n}A_{i}}\;i\neq s,$$

where the sum in the denominator is over the rest of the *A*_*i*_ oscillator amplitudes. Notice that this measure of consistency should be negatively correlated with the entropy measure of Desain and Honing: As the signal gets weaker relative to the noise, the entropy of the participants' choices of category should go up.

**Figure 4.**
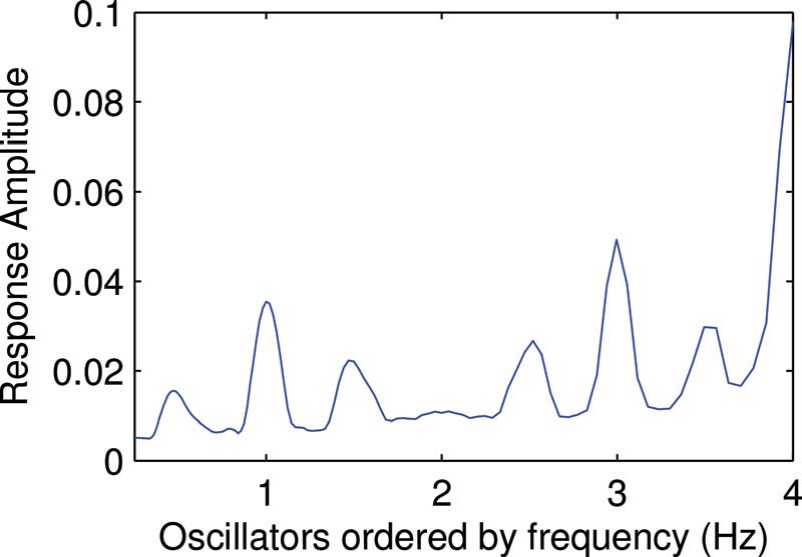
An example of an activation pattern generated by feeding the resonance model a rhythm with IOIs 0.25 s, 0.5 s, and 0.25 s.

Prediction (B) implies that rhythm sequences resulting in similar states when given as input to a resonance model should be categorized similarly in an experimental task. A resonance model does not directly produce a categorization, but this is not required for testing this prediction. It is possible to compare the resulting states of two rhythm sequences by calculating the respective activation patterns and comparing these. A suitable similarity measure is given by considering the activation patterns as points in an *n*-dimensional space, where *n* is the number of oscillators in the resonance model, and then taking the Euclidean distance between these two points, where a shorter distance corresponds to more similar states. Considering the twelve most common rhythm categories chosen by the participants in Desain and Honing's study as prototype categories, it is possible to use the rhythm sequences corresponding to these categories to generate *prototype activation patterns*. For example, to generate the prototype activation pattern for the category 1-2-1 (as shown in Figure 4) the rhythm sequence with IOIs 0.25 s, 0.5 s, and 0.25 s would be used as input to the resonance model. A rhythm sequence's activation pattern can then be compared with these prototypes' activation patterns and the prototype category with the most similar activation pattern can be assigned to that rhythm sequence. In this way, all rhythm sequences can be assigned a category and these categories can be compared with the categories selected by the participants in Desain and Honing's study. Specific hypotheses are then that a resonance model categorization of the stimulus used by Desain and Honing should be similar to the categorization made by the participants in the no meter, duple meter, and triple meter conditions. Furthermore, as the participants' categorizations of the rhythm sequences in the no meter condition were more similar to the categorizations made in the duple meter condition than to the categorizations made in the triple meter condition, the same relation should be present in the categories generated by the resonance model.

## 2 Methods

**Figure 5.**
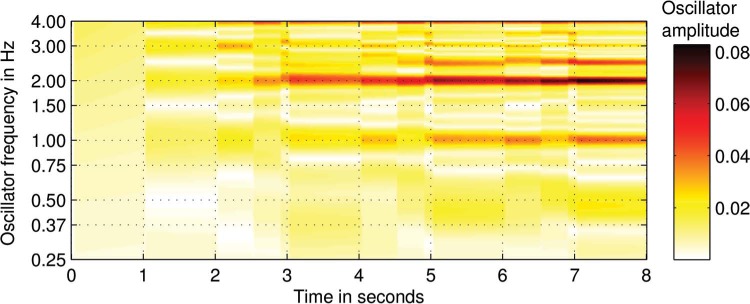
An example of oscillator activation over time for an oscillator network given the rhythm pattern [0.5, 0.375, 0.125].

The resonance model was implemented in MATLAB (http://www.mathworks.se/products/matlab/) using the Nonlinear Time-Frequency Transformation Workbench (Large & Velasco, in preparation). The model consisted of 145 *Hopf oscillators*, a type of oscillator that entrains to periodic input and where the amplitude of an oscillator depends on that oscillator's intrinsic frequency and the periodicities of the input. The differential equation of the Hopf oscillator used is:

(3) $$\begin{array}{c} \frac{\mathrm{dz}}{\mathrm{dt}}=z(\alpha +i\omega +\frac{\beta \varepsilon {\left|z\right|}^{4}}{1-\varepsilon {\left|z\right|}^{2}})+\frac{x}{1-\sqrt{\varepsilon }x}\cdot \frac{1}{1-\sqrt{\varepsilon z}}\\ \;\alpha =-0.1,\beta =-0.1,\varepsilon =0.5 \end{array}$$

where *α* is a damping term, *β* is an amplitude compression factor and *∊* is a scale factor. The last term in Equation (3) is the resonant term, which is dependent on the stimulus *x*. These parameter values and this specific formulation of the Hopf oscillator were not chosen on the basis of any specific theoretical considerations (see Section 4 for a discussion of these choices); many other configurations are possible and a more general form of the Hopf oscillator is derived in Large, Almonte, and Velasco (2010). The oscillators' intrinsic frequencies were centered around 1 Hz with frequencies logarithmically distributed from 0.25 Hz to 4 Hz. Figure 5 shows an example of the activation over time for this network of oscillators given the rhythm pattern [0.5, 0.375, 0.125]. The method used for creating activation patterns was that in Equation (1) with *t*_*s*_ set to the time step corresponding to half the stimulus length and *t*_*e*_ set to the last time step. The MATLAB code for the model and both input data and the resulting output are available on request from the first author. The code for the Nonlinear Time-Frequency Transformation Workbench (Large & Velasco, in preparation) has not yet been publicly released and has to be requested separately.

The 66 rhythm sequences from the no meter condition were encoded and given as input to the model yielding 66 activation patterns. In accordance with Desain and Honing (2003), the repeated rhythm pattern in each sequence was 1-s long and repeated three times, creating an eight bar long sequence of where the third, fifth, and seventh bar contained the rhythm pattern. The IOIs in the sequences range from 3/19 s to 13/19 s in steps of 1/19 s, creating the grid seen in Figures 2 and 3. This was repeated for the sequences from the duple and triple meter conditions. Additionally, the sequences of the prototype categories were encoded in the same way as in the no meter condition sequences, yielding 12 prototype activation patterns.

### Analysis

The signal-to-noise ratio, defined by Equation 2, was calculated for each of the 66 rhythm sequences in the no meter condition. Here a higher value represents a stronger signal and the signal-to-noise ratio is taken as a measure of how easy it is to classify the corresponding sequence. For each of all 198 rhythm sequences, the Euclidean distance to each of the twelve prototype sequences was calculated. A sequence is categorized as the prototype it is closest to in the Euclidean space, with each dimension representing one oscillator in the oscillatory network. The resulting categories for the no meter condition can be seen in Figure 3.

Randomized permutation tests (Ernst, 2004) were used to compare the categorization of the rhythm sequences from the behavioral data with the categorization from the resonance model. Given two different categorizations of the 66 rhythms, a similarity score is calculated as the number of rhythms that are given the same category by both categorizations. In the cases where the most common categorization of a specific rhythm sequence in the behavioral data is not one of the 12 prototype categories this rhythm sequence is excluded from further analysis. Next, all category labels are randomly reassigned to the rhythm sequences and a new similarity score is calculated. This is repeated 10,000 times, yielding a randomized permutation distribution of similarity scores. This is the distribution that is expected under the null hypothesis that there is no relation between the categorization by the model and the categorization by the participants. A *p*-value is then calculated as the probability of achieving the actual similarity score, or a more extreme similarity score, given the distribution of randomized similarity scores. The permutation tests were two-tailed (calculated according to the method in Ernst, 2004) in all cases except where noted.

## 3 Results

The signal-to-noise measure was calculated for all activation patterns in the no meter condition and, as hypothesized, a negative correlation between Desain and Honing's (2003) entropy measure of consistency and the signal-to-noise ratio was found (Pearson product-moment correlation, *r* = −0.32, *p* = 0.009). These two measures of consistency are expected to have an inverse relationship, that is, low entropy in the experimental data indicates high consistency, while a low signal-to-noise ratio in the simulated data indicates low consistency. A comparison between these two measures of consistency for the experimental and the simulated data is shown in Figure 2. To facilitate comparison, the color scales have been matched so that red indicates low consistency while blue indicates high consistency. The measures of consistency are comparable, showing the same broad patterns in both the simulated (Figure 2b) and experimental data (Figure 2a).

The activation patterns for all of the three metrical conditions were compared with the prototype activation patterns using the Euclidean distance as the similarity measure and each rhythm sequence was assigned the category of the most similar prototype. A comparison with the categories assigned in the experimental task for the no meter condition is shown in Figure 3. The categorizations agree to a large extent. The 1-1-1 category is the most common in both the experimental and the simulated categorizations and both categorizations exhibit roughly convex category regions. Here convexity refers to the property that for every pair of points within a geometric object there exists a line, also within the object, that connects the points. The convexity of the category regions accords with Gärdenfors' (2000) general prediction for category representations. A randomized permutation test also showed that the categorization generated by the resonance model and the categorization from Desain and Honing's data were more similar than would be expected by chance alone for all three of the metrical conditions. In the no meter condition (shown in Figure 3) the agreement was 71% (*p* < 0.001) and in the duple and triple meter conditions 67% (*p* < 0.001) and 61% (*p* < 0.001), respectively.

**Figure 6.**
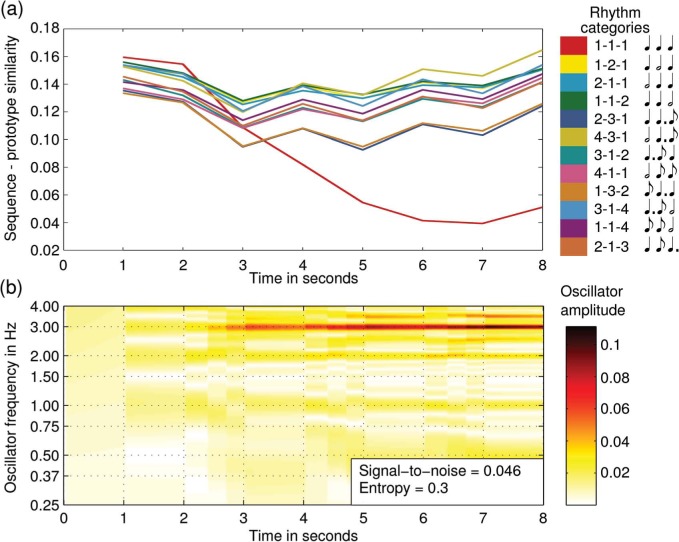
The oscillator activation and corresponding categorization over time for an oscillator network given a rhythm pattern that scored low entropy in Desain and Honing (2003).

**Figure 7.**
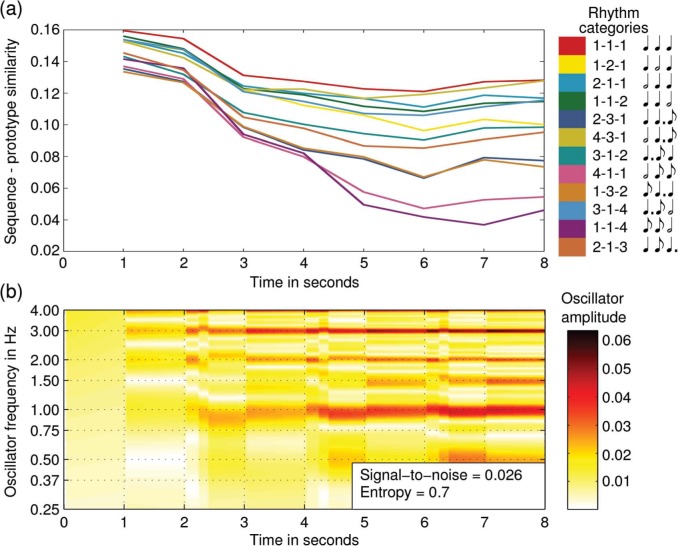
The oscillator activation and corresponding categorization over time for an oscillator network given a rhythm pattern that scored high entropy in Desain and Honing (2003).

Figure 6 shows the oscillator activation over time and the corresponding dynamic categorization given a rhythm sequence that was assigned low entropy in Desain and Honing's data. Compare this with Figure 7 that shows the oscillator activation and dynamic categorization for a rhythm that was assigned high entropy in Desain and Honing's data. For the low entropy rhythm the signal-to-noise ratio is high and the categorization is more stable. For the high entropy rhythm, however, the signal-to-noise ratio is low and the categorization in never stable, that is, there never emerges one clear winner.

In the experimental data, the categorization of the duple meter condition was more similar to the no meter condition than was the triple meter condition, and this was also the case for the simulated categorizations. The agreement between the no meter condition and the duple and triple meter conditions for the simulated categorizations was calculated as being 77% and 71%, respectively with duple meter agreeing with the no meter categorization in 6 percentage points more of the cases (*p* = 0.045, one-tailed randomized permutation test).

## 4 Discussion

Many models of categorical perception have been based on neural networks and there exist several models of rhythm perception based on neural networks (Desain & Honing, 1989; Miller, Scarborough, & Jones, 1992; Mozer, 1993). We believe that using a dynamical system of resonating oscillators provides a physiologically more plausible way of modeling such phenomena. By modeling rhythm perception in such a system, we have shown that it is possible to explain empirical findings of listeners' categorical perception of rhythm. Our oscillator model has been able to accurately replicate the experimental data from Desain and Honing (2003). A possible concern is whether the model is sensitive to the choice of parameters. However, a parameter sensitivity analysis has not been performed as the purpose of the model is not to predict the experimental data as well as possible nor do we claim that the specific model configuration could not be the subject of improvements. What is claimed is that the model supports the notion that resonance theory is a viable model of rhythm perception and that by viewing rhythm perception as a dynamical system it is possible to model properties of rhythm categorization.

An advantage of oscillator models is that they can be generalized to other kinds of categorical perception. Examples from the domain of music are pitch perception and tonality perception (Large, 2010). Oscillatory models are not confined to temporal processes and can be used for other modalities. The main importance of our model is perhaps that the example of how oscillator models can be constructed for categorical rhythm perception can serve as inspiration for similar models of other cognitive phenomena. A general question is whether the convexity of rhythm categories generated from our model generalizes to other areas of categorical perception when oscillator models are used. If so, it could be interpreted as a general mechanism that can explain the convexity of categories as put forward by Gärdenfors (2000).
